# Cost-effectiveness analysis of second-line medical therapies in acromegaly: a real-life study

**DOI:** 10.3389/fendo.2025.1573721

**Published:** 2025-04-28

**Authors:** Eva Venegas Moreno, Andrés Jiménez-Sánchez, Pablo Remón-Ruiz, Elena Dios, Jaime Perea Cortés, Celia Hernández-Reina, David A. Cano, Alfonso Soto Moreno

**Affiliations:** ^1^ Unidad de Gestión Clínica de Endocrinología y Nutrición, Hospital Universitario Virgen del Rocío, Seville, Spain; ^2^ Instituto de Biomedicina de Sevilla, IBiS/Hospital Universitario Virgen del Rocío/CSIC/Universidad de Sevilla, Seville, Spain

**Keywords:** acromegaly, pasireotide, pegvisomant, cost analysis, radiotherapy

## Abstract

**Introduction:**

Acromegaly is an uncommon disease with important comorbidity and economic cost. Although the pharmacological cost of second-line treatment for refractory acromegaly has been theoretically analyzed, real-life studies are needed.

**Objectives:**

To assess the use of pasireotide and pegvisomant in a third-level center under routine clinical practice.

**Methods:**

Acromegaly patients that had been treated with pasireotide and/or pegvisomant were included in (A) a cross-sectional study (two years after starting these drugs) to analyze the cost of acromegaly, hormone replacement, and type 2 diabetes mellitus (T2DM) treatments, and the cost of surgery and radiotherapy; and (B) a retrospective cohorts study (May 2006—October 2024) to analyze efficacy, safety (adverse events, fasting glucose, glycated hemoglobin, and T2DM diagnosis), and dose evolution. Descriptive statistics were 10% trimmed means and standard deviation. Two-tailed hypothesis testing with Yuen’s t and Fisher’s test had a *P* < 0.05 significance.

**Results:**

25 participants were included in the transversal study and 31 participants in the longitudinal study. A typical patient with a poorly granulated GH-producing adenoma underwent in-center surgery once and received radiotherapy. In the transversal study, total pharmacological cost was 34,139.29 (13,472.09) €/person/year, with 33,874.88 (13,468.36) €/person/year for second-line acromegaly drugs. Pasireotide displayed 9,423.26 €/person/year worth of savings (*P* = .12), reaching 30,415.98 €/person/year at high dose (*P* < 0.001). In the longitudinal study, pasireotide dose was reduced (*P = .*06) regardless of treatment modality. Pasireotide affected carbohydrate metabolism (*P* = .001), but the effect was generally mild.

**Conclusions:**

Pasireotide was found to be a more cost-effective option in patients with first-line treatment failure.

## 1 Introduction

Acromegaly is a low-prevalence endocrine disease (1, 2) caused by excess growth hormone (GH) production in the pituitary, in most cases secondary to a benign tumor (adenoma) of somatotropic cells.

Early treatment of this condition is of paramount importance, since acromegaly remains a serious condition because it causes comorbidities with important health repercussions (3–7). Biochemical control of the disease can normalize life expectancy (8), reduce the incidence of comorbidities (9, 10), and improve patient-reported quality of life (11).

Whenever feasible, pituitary surgery is the first line of treatment (12), with cure rates via a transsphenoidal approach ranging from 47.6% to 76.4% (13). Refractory cases may require adjuvant radiotherapy or radiosurgery. Drug treatment may be required preoperatively in select patients, or a posteriori in cases not cured after surgery. Drug therapy consists of first-generation synthetic somatostatin analogs (SSAs) octreotide LAR and lanreotide, second-generation somatostatin analogs (pasireotide), GH receptor antagonists (pegvisomant), and dopamine agonists (cabergoline). First-generation SSAs are considered first-line, while all other drugs are considered second-line therapies and are used if biochemical control or remnant tumor control proves insufficient, or if the first-line drug is not tolerated (12, 14).

According to randomized clinical trials, pegvisomant and pasireotide are the most effective options, with a probability of disease control of 73.4% and 73.0%, respectively (15). Regarding its safety, most studies report injection site reactions and gastrointestinal disorders that range from mild to moderate (15).

From an economic viewpoint, a previous study (16) has analyzed the cost-efficacy of pegvisomant and pasireotide in refractory acromegaly in the Spanish national health system, using a Markov-type model that was based on clinical trials data. Although it is a valuable contribution, this work could be considered a theoretical exercise. Therefore, its conclusions may differ from those obtained in a routine clinical practice setting.

To our knowledge, no previous publication has evaluated the cost of the different second-line drug options for acromegaly in a real-life setting. The aim of our study was to ascertain current and real data on the efficacy, safety and cost of all second-line medical treatment modalities for acromegaly, both as monotherapy and in combination therapy, in patients with refractory acromegaly treated in routine clinical practice conditions in a hospital classified as a reference center (Centros, Servicios y Unidades de Referencia [“CSUR”]) for hypothalamic-pituitary diseases. The main working hypotheses were (1): the cost of pegvisomant and pasireotide might not be similar; and (2) treatment with pasireotide may allow for de-escalation during follow-up.

## 2 Methods

### 2.1 Design

A single-center, analytical observational study conducted at Hospital Universitario Virgen del Rocío (Seville, Spain). This manuscript was prepared following the STROBE recommendations (17).

**Figure 1:**
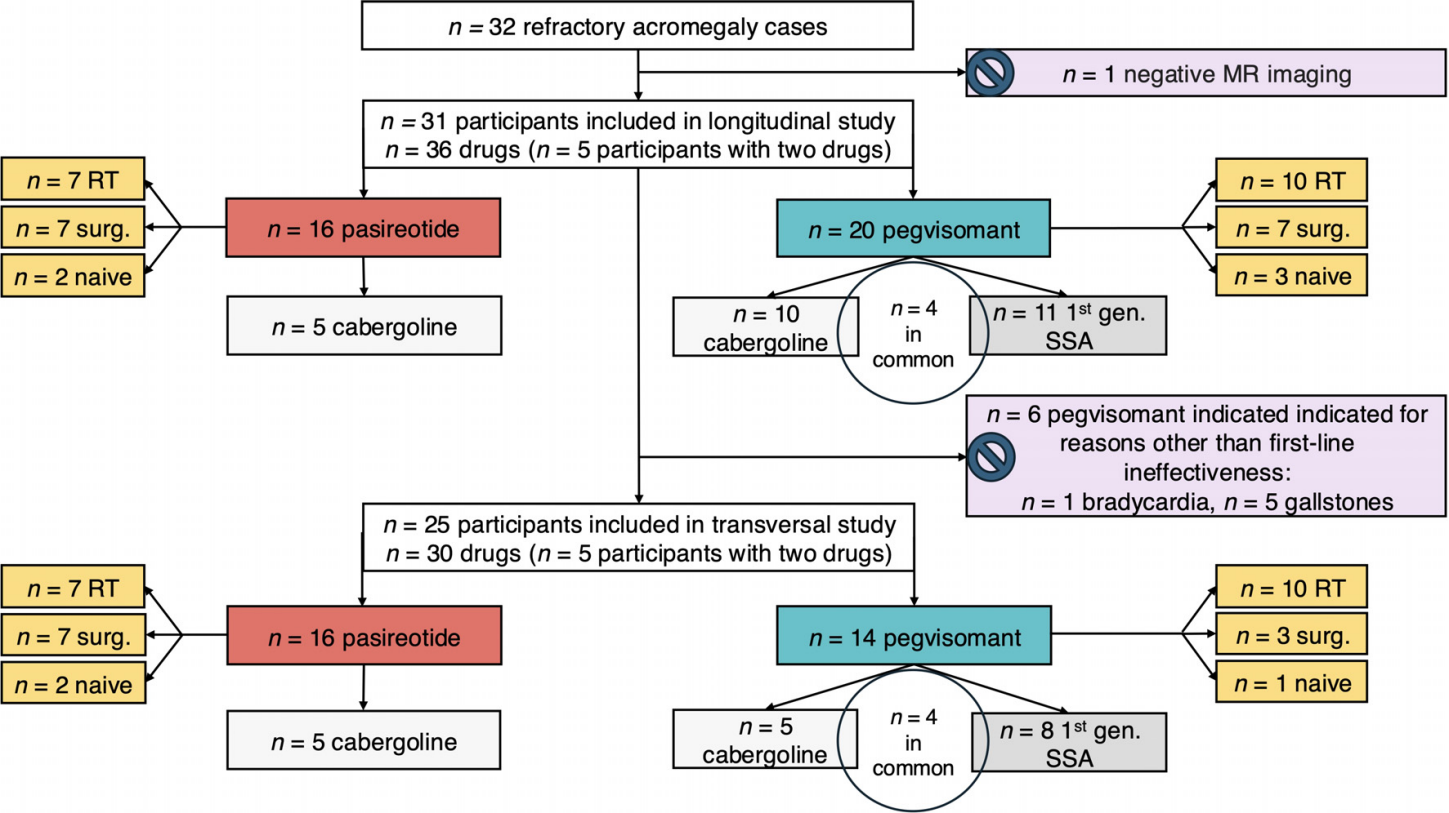
Flow diagram of the participants. Our whole cohort of refractory acromegaly cases is represented in the upper rectangle. From it, participants for the longitudinal study are extracted after completing the inclusion and exclusion criteria from **Table 1**, with an excluded case depicted in a pink rectangle that horizontally diverges from the top-to-bottom direction of the figure. Then, participants for the transversal study are extracted after completing the inclusion and exclusion criteria from **Table 1**, with *n* = 6 excluded cases depicted in a pink rectangle that horizontally diverges from the top-to-botton direction of the figure. In all cases, pasireotide treatment is represented in red and pegvisomant treatment in green. Coadjuvant treatments such as cabergoline and first generation synthetic somatostatin analogues (1^st^ gen. SSAs) are represented in subsequent rectangles in different tones of grey, with participants taking both kind of coadjuvant drugs represented within a tangent circle.

The study consists of two sub-studies (**Figure 1**) whose inclusion and exclusion criteria are shown in **Table 1**.

**Table 1:** Inclusion and exclusion criteria of the transversal study (cross-sectional design) and the longitudinal study (cohorts design).

	Longitudinal study	Transversal study
Inclusion	(1) Patients over 18 years of age diagnosed with acromegaly (2) Patients requiring drug treatment with one or more second-line drugs (pasireotide and/or pegvisomant), together with or without first-generation somatostatin synthetic analogs (SSAs) or cabergoline concomitantly for the control of acromegaly, and regardless of having previously received other therapeutic modalities (surgery and/or radiotherapy).	(1) Patients over 18 years of age diagnosed with acromegaly (2) Patients requiring drug treatment with one or more second-line drugs (pasireotide and/or pegvisomant), together with or without first-generation somatostatin synthetic analogs (SSAs) or cabergoline concomitantly for the control of acromegaly, and regardless of having previously received other therapeutic modalities (surgery and/or radiotherapy). (3) Two years on treatment with one or more second-line drugs.
Exclusion	(1) No compatible pituitary imaging.	(1) No compatible pituitary imaging. (2) The indication of pegvisomant was not due to inefficacy of first-line treatment. (3) Poor disease control at that time (defined as normal levels of insulin-like growth factor type 1 [IGF-1] according to our reference laboratory - see **Supplementary Table 1**)

The longitudinal study involves a retrospective cohort design to analyze the safety and efficacy of second-line drugs for the treatment of acromegaly, regardless of their indication (poor response, intolerance or contraindication to first-line treatment). Data was collected from the date of start of the first treatment on 5 May 2006 to 10 October 2024. The transversal study involves a cross-sectional design to analyze pharmacological (second-line drugs and adjuvants for the treatment of acromegaly, hormone replacement therapy, type 2 diabetes mellitus [T2DM]) and non-pharmacological costs (surgery, radiotherapy), and only includes patients with an indication of second-line drug due to poor response to first-line treatment already controlled with the second-line drug (defined as normal levels of insulin-like growth factor 1 [IGF-1], according to our reference laboratory - see **Supplementary Table 1**). Data was collected from the visit closest in time to the two years of follow-up after the start of the second-line drug, since in our experience this is the time at which drug titration is completed and patients reach a stable dose. Treatment was indicated according to standard clinical practice and protocolized by our center.

### 2.2 Data collection

The data was obtained from electronic health records. Age, sex, identifiers, date of diagnosis of acromegaly, date of start of treatment with surgery and radiotherapy, days of admission and complementary tests associated to surgery and radiotherapy, presence of major and minor complications attributable to pegvisomant or pasireotide, dates of admission and discharge due to major complications, diagnostic and therapeutic tests required due to major complications, diagnosis of T2DM (based on clinical history) or prediabetes following ADA criteria (considered as glycated hemoglobin [HbA1c] 5.7-6.4% and/or a fasting blood glucose 100.0 - 126.0 mg/dL), blood glucose control markers (fasting plasma glucose [Glu], HbA1c) and disease control markers (IGF-1), drug treatment (second-line drugs for acromegaly, hormone replacement therapy, and antidiabetic treatment) were recorded.

### 2.3 Cost calculation

Local costs for acromegaly drugs (**Table 2**), hormone replacement therapy (**Supplementary Table 2**), and antidiabetic treatment (**Supplementary Table 3**) were obtained from electronic health records. The sum of all these costs has been defined as the total pharmacological cost. Costs according to each participant are expressed as euros per person per year (€/person/year), estimating a 360-day year (equivalent to 12 30-day months). Acromegaly co-treatment cost has also been included: both the cost of pasireotide and pegvisomant include the cost of cabergoline, and the cost of pegvisomant also includes the cost of first-generation SSAs. Calculations were performed based on drug dose at the cross-sectional timepoint in the transversal study. We also performed sub-analyses, dividing the sample according to second-line acromegaly drug dose: low dose was defined as any dose of 20 mg/month for pasireotide and ≤ 105 mg/week for pegvisomant. The rationale behind this cut-off point for pegvisomant is that it was the median dose after 3 to 7 years of treatment in Spain in ACROSTUDY (18).

**Table 2:** Cost of drugs for acromegaly.

Drug	Milligrams	Units per container	Cost per container (€)	Cost per unit (€)
Cabergoline (Teva)	0.5	2	4.14	2.07
	0.5	8	16.52	2.06
	1	20	13.77	0.69
	2		27.54	1.38
Lanreotide (Myrelez®/Somatuline® autogel)	60	1	371.50	371.50
	90		478.31	478.31
	120		558.39	558.39
Octreotide LAR (Sandostatin® LAR)	10	1	192.10	192.10
	20		341.65	341.65
	30		486.00	486.00
Pasireotide (Signifor®)	20	1	2,481.35	2,481.35
	40		2,554.15	2,554.15
	60		2,779.83	2,779.83
Pegvisomant (Somavert®)	10	30	2,079.28	69.30
	15		3,089.85	102.99
	20		4,100.42	136.68
	25		5,110.99	170.37
	30		6,121.55	204.05

The dose of drugs has been expressed in milligrams or international units, and the administration time is expressed in days, week or month, depending on the type of drug.

All direct non-drug costs (pituitary surgery, pituitary radiotherapy, major adverse events) in the transversal study have been calculated according to the updated prices in Andalusia included in the public price catalogue of health services provided by the Andalusian Health Service (19) (**Table 3**). In this case, the undivided unit cost per year (€/person) has been calculated. To calculate the cost of the major adverse events, we considered the days of admission, the complementary tests requested, and the treatments required for said complications. We did not count the cost of visits to Endocrinology or the cost of imaging tests during follow-up, since we assumed this to be constant in all participants. Likewise, we have not counted the direct non-pharmacological cost attributable to T2DM (such as therapeutic education sessions or screening for T2DM complications).

**Table 3:** Cost of hospital admissions and associated diagnostic and therapeutic procedures.

Description	Cost per unit (€)
Pathology: Pituitary biopsy or surgical specimen	73.36
Pathology: Conventional histochemical techniques	18.34
Pathology: Cytogenetics in solid tumors	275.10
Pathology: Electron microscopy	458.50
Radiodiagnosis: Computed tomography without contrast	55.38
Radiodiagnosis: Computed tomography with contrast	119.99
Radiodiagnosis: Magnetic resonance imaging with and without contrast	323.05
Radiodiagnosis: aortic arch and supra-aortic trunk angiography	443.04
Ophthalmology: Campimetry	161.55
Radiation Oncology: Radical radiation therapy of the pituitary gland	3,137.19
Radiation Oncology: Radical radiation therapy of the pituitary gland in linear accelerator	3,679.22
Neurosurgery: Lumbar puncture for CSF study	306.61
Neurosurgery: Conventional hospitalization due to nervous system neoplasms with surgery	8,298.55
Neurosurgery: day of admission	635.65
Gastrointestinal surgery: Conventional hospitalization due to cholecystectomy with biliary tract examination	9,461.38
Gastrointestinal surgery: Conventional hospitalization due to cholecystectomy without biliary tract examination	7,906.53
Gastrointestinal surgery: Conventional hospitalization due to laparoscopic cholecystectomy without biliary tract examination	6,138.22
Gastrointestinal surgery: Conventional hospitalization due to laparoscopic cholecystectomy with biliary tract examination	6,724.96
General and gastrointestinal surgery: day of admission	603.70
Gastroenterology: conventional hospitalization due to pancreatic disorders, except malignancy	3,822.10
Gastroenterology: diagnostic endoscopic retrograde cholangiopancreatography (ERCP)	559.69
Gastroenterology: diagnostic-therapeutic ERCP	1,019.84
Gastroenterology: day of admission	376.24
Internal Medicine: day of admission	324.01
Endocrinology/Internal Medicine: conventional hospitalization due to diabetes with age > 35 years	3,658.24
Endocrinology and nutrition: day of admission	1,010.88
Radiodiagnosis: Ultrasound	36.92

### 2.4 Data analysis

All participants were included for analysis using the software RStudio version 2023.06.1 + 524 (2023.06.1 + 524) and *cowplot* (20), *ggpubr* (21), *ggstatsplot* (22), and *tidyverse* (23) packages. After assessing the presence of normal data distribution with the Shapiro-Wilk test, we used a mean trimmed to 10% (standard deviation) for non-normal distributions. Comparisons between trimmed means were performed using a robust test (Yuen’s t-test), and comparisons between proportions were performed using Fisher’s exact test. Some comparisons have been made via intention-to-treat analysis (ITT), analyzing all participants who started a second-line drug. Other comparisons have been made per protocol (PP), analyzing only those who have chronically maintained treatment. All hypothesis testing was performed on a two-tailed basis, with statistical significance being considered for *P* <.05.

## 3 Results

### 3.1 Clinical-demographic description of the sample

A total of *n* = 31 participants were enrolled in the longitudinal study and *n =* 25 in the transversal study (**Figure 1**).

The clinical-demographic characteristics of the participants in both sub-studies are shown in **Table 4**.

**Table 4:** Clinical-demographic description of the cross-sectional sample.

Parameter	Transversal study	Longitudinal study
Sample size	*n = *25 participants *n = *30 drugs	*n = *31 participants *n = *36 drugs
Sex		
Men Women	*n = *14 men *n = *11 women	*n = *20 men *n = *16 women
Current age (years)	48.8 (13.5)	50.9 (13.7)
Age at diagnosis (years)	41.7 (12.9)	43.2 (13.7)
Time since onset (years)	6.4 (4.5)	6.4 (4.9)
Surgery		
Yes No	*n = *22 *n = *3	*n = *26 *n = *5
Number of surgeries		
1 2	*n = *19 *n = *3	*n = *21 *n = *5
Surgery at external site		
Yes No	*n = *6 *n = *16	*n = *6 *n = *20
Radiotherapy		
Yes No	*n = *14 *n = *11	*n = *14 *n = *17
Therapeutic modality		
Surgery only Surgery and radiotherapy None	*n = *8 *n = *14 *n = *3	*n = *12 *n = *14 *n = *5
Immunostaining*		
GH GH + PRL GH + TSH	*n = *11 *n = *9 *n = *0	*n = *12 *n = *11 *n = *1
Granule density**		
Low Dense	*n = *12 *n = *2	*n = *14 *n = *3
Type 2 diabetes mellitus diagnosis***		
Yes No	*n = *12 *n = *8	*n = *13 *n = *18
Type 2 diabetes mellitus treatment		
DPP4i GLP1Ra basal insulin ultrarapid insulin metformin SGLT2i sulfonylurea	*n* = 1 *n = *0 *n = *2 *n = *2 *n = *12 *n = *2 *n = *1	*n = *2 *n = *2 *n = *1 *n = *0 *n = *15 *n = *2 *n = *2

*n = 2 participants at external site did not provide immunostaining results.

**n = 8 participants did not have granule density in their pathology report.

***In the case of the longitudinal study, this refers to diagnosis of T2DM at the end of the follow-up period.

In the transversal study, 14 patients received radiotherapy. Only *n =* 3 participants required two pituitary surgeries (all of them also received radiation therapy, and one of them received two second-line drugs), and *n =* 3 participants did not receive surgery or radiation therapy (treated with second-line drugs only). In turn, *n =* 4 participants received both cabergoline and a first-generation SSA.

### 3.2 Pharmacological cost: second-line drugs for the treatment of acromegaly

**Figure 2:**
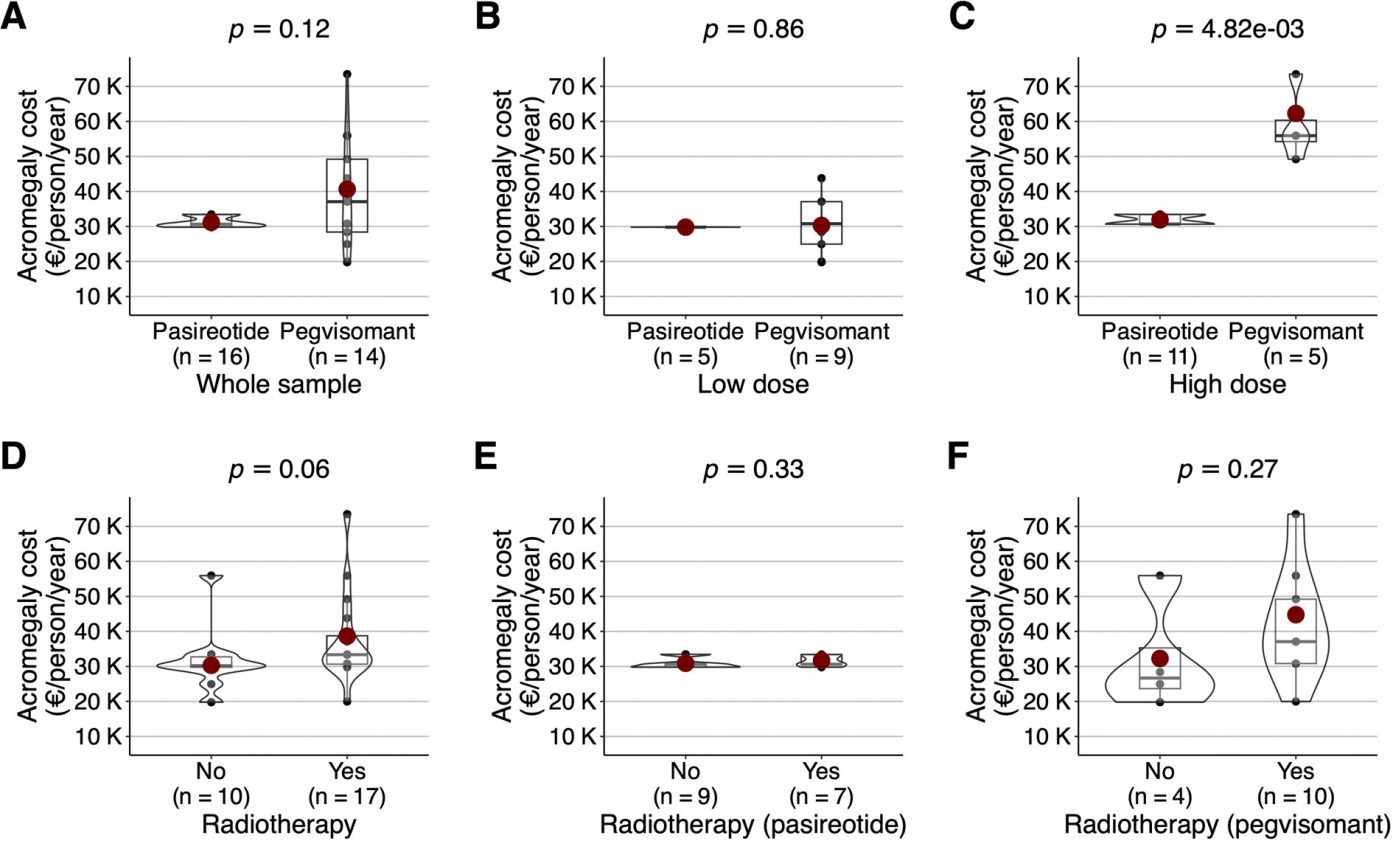
Box and whisker plot (with 10% trimmed mean represented as a reddish dot) with overlapping violin plot and point cloud diagram geometries. *P*-values of the robust test for comparisons of trimmed means (Yuen’s t test) are shown at the top. Annual cost per person (expressed in €/person/year, with each thousand units replaced by factor *k*) of second-line acromegaly drugs on the *Y-*axis in all cases. Type of second-line acromegaly drugs (pasireotide or pegvisomant) on the *X-*axis in the overall sample **(A)**, in patients on low-dose **(B)**, and on high-dose **(C)** treatment. Presence of radiotherapy on the *X-*axis in the overall sample **(D)**, in patients treated with pasireotide **(E)**, and with pegvisomant **(F)**.

The dose and pharmacological cost of second-line acromegaly drugs followed a non-normal distribution. Cost was 33,874.88 (13,468.36) €/person/year. Pasireotide dose was 40.0 (16.3) mg/month, with a cost of 31,194.04 (1,544.98) €/person/year. Pegvisomant dose was 107.1 (52.1) mg/week, with a cost of 40,617.30 (18,409.04) €/person/year. The cost difference was 9,423.26 €/person/year in favor of pasireotide (*P* = .12) (**Figure 2A**).

In the low-dose group, pasireotide dose was 20.0 mg/month, and its cost was 29,796.04 (44.37) €/person/year. Pegvisomant dose was 78.3 (21.1) mg/week, and its cost was 30,293.49 (8,089.30) €/person/year. The cost difference was 497.45 €/person/year in favor of pasireotide (*P* = .86) (**Figure 2B**). In the medium-high dose group, pasireotide dose was 48.9 (10.4) mg/month, and its cost was 31,873.64 (1,431.37) €/person/year. Pegvisomant dose was 168.0 (38.3) mg/week, and its cost was 62,289.62 (12,160.16) €/person/year. The cost difference was 30,415.98 €/person/year in favor of pasireotide (*P* <.001) (**Figure 2C**).

Radiotherapy was associated with a greater cost in second-line acromegaly drugs, in comparison with surgery without radiotherapy: 38,667.13 (15,653.40) vs 30,313.39 (9,394.76) €/person/year, with an excess cost of 8,353.74 €/person/year (*P = .*06) (**Figure 2D**). In the radiotherapy group, pasireotide dose was 45.7 (15.1) mg/month, and costed 31,695.09 (1,606.23) €/person/year (**Figure 2E**); while pegvisomant dose was 118.1 (55.3) mg/week, and costed 44,767.66 (18,618.69) €/person/year (**Figure 2F**). The cost difference was 13,072.57 €/person/year in favor of pasireotide (*P = .*09). For participants requiring two surgical interventions, cost was 30,213.00 (617.73) €/person/year for pasireotide and 55,283.92 (25,749.32) €/person/year for pegvisomant (*P* = .40).

Cabergoline co-treatment did not significantly change second-line acromegaly drugs cost. In pasireotide, participants with cabergoline had higher doses (48.0 vs 35.5 mg/month, *P* = .24) and costs (32,176.04 vs 30,659.51 €/person/year, *P* = .14). In pegvisomant, those treated with cabergoline required a higher dose (122.5 vs 91.2 mg/week, *P* = .46) and therefore had higher cost (48,018.38 vs 37,611.22 €/person/year, *P* = .49).

In pegvisomant, co-treatment with first-generation SSAs did not significantly change second-line acromegaly drugs cost, being 42,940.00 vs. 40,245.77 €/person/year (*P* = .81). This effect appeared independently of pegvisomant dose, as cost was 62,917.28 vs. 61,348.12 €/person/year (*P* = .92) in the low-dose pegvisomant group, and 30,753.31 vs. 29,718.72 €/person/year (*P* = .86) in the high-dose pegvisomant group.

### 3.3 Total drug cost

**Figure 3:**
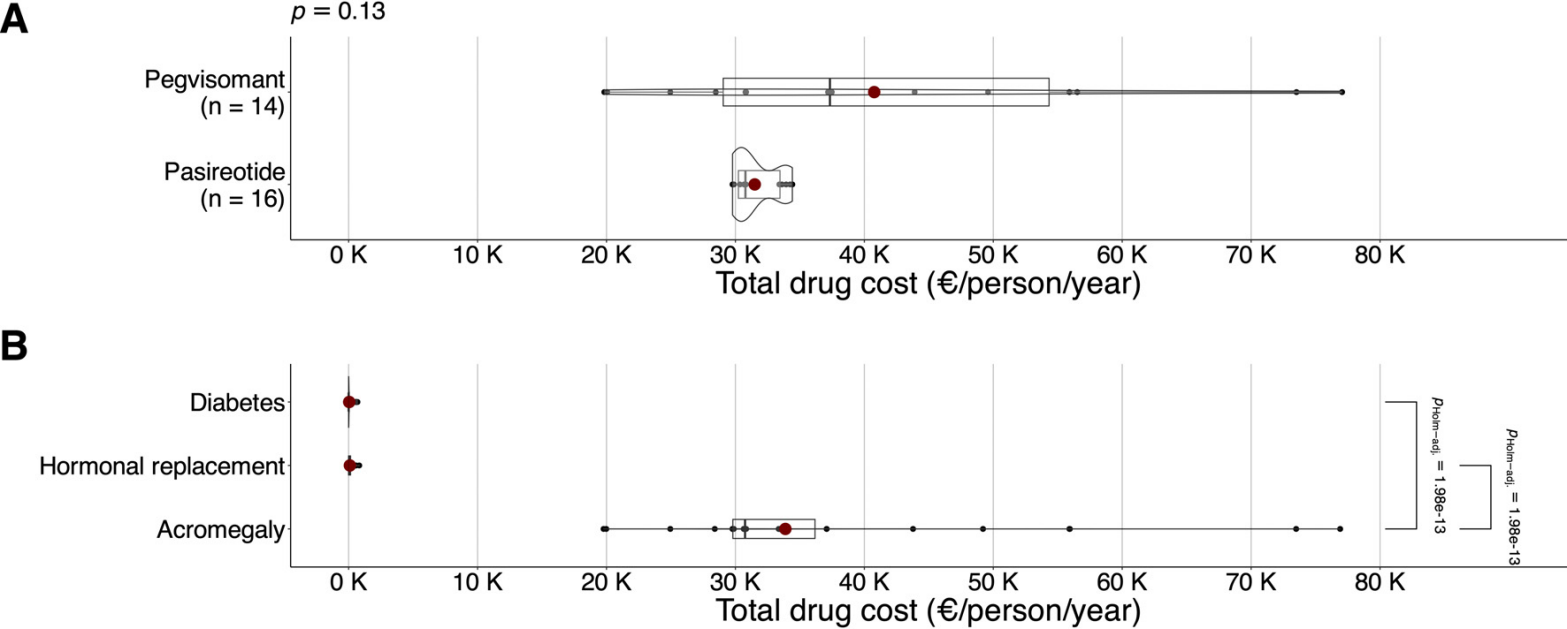
Box and whisker plot (with 10% trimmed mean represented as a reddish dot) with overlapping violin plot and point cloud diagram geometries. Total annual drug cost per person (expressed in euros/person/year, with each thousand units replaced by factor *k*) on the *X*-axis according to second-line acromegaly drugs on the *Y*-axis **(A)**, with *p*-values of the robust test for comparisons of trimmed means (Yuen’s t test) shown at the top, and broken down by type of cost (treatment of diabetes, hormone replacement therapy, treatment of acromegaly) in the sample on the *Y*-axis **(B)**.

The total drug cost followed a non-normal distribution, being 34,139.29 (13,472.09) €/person/year in the sample, 31,499.99 (1,729.66) €/person/year for pasireotide, and 40,765.59 (18,456.28) €/person/year for pegvisomant (*P* = .13) (**Figure 3A**). Second-line acromegaly drugs displayed a higher cost than hormone replacement (*P* <.001) and T2DM (*P* <.001) (**Figure 3B**).

The cost of hormone replacement followed a non-normal distribution, being 95.45 (214.21) €/person/year. Stratifying by second-line acromegaly drugs, it was 108.738 (247.63) €/person/year for pasireotide, and 112.042 (177.65) €/person/year for pegvisomant (*P* = .97). Radiotherapy associated a higher cost in hormone replacement therapy: 172.12 (236.14) vs 25.92 (€59.79) €/person/year (*P* = .02).

The pharmacological cost of T2DM followed a non-normal distribution, being 205.33 (278.62) €/person/year. According to second-line acromegaly drugs, it was 243.76 (289.99) €/person/year for pasireotide, and 32.40 (15.27) €/person/year for pegvisomant, with a difference of 211.36 €/person/year in favor of pegvisomant (*P = .*02).

### 3.4 Cost of surgery and radiotherapy

According to hospital protocol, all admissions involved a post-surgical MRI scan with and without contrast, as well as an immunohistochemical study of the surgical specimen. The duration of admission was 8 (5) days. Taking this data into account, the cost of pituitary surgery was 14,526.37 (5,939.35) €/person. Regarding radiotherapy, its cost was 3,137.00 €/person in all cases.

### 3.5 Dose evolution of second-line drugs for the treatment of acromegaly (pasireotide and pegvisomant)

A total of *n* = 31 participants in the longitudinal study received *n* = 16 pasireotide, and *n* = 20 pegvisomant (**Figure 1**). Second-line therapy for acromegaly was discontinued on *n* = 16 occasions (*n* = 4 for pasireotide, *n* = 12 for pegvisomant). Discontinuation due to adverse events occurred only in the case of pasireotide (*n* = 2). Withdrawal due to non-treatment-related death occurred both in the case of pasireotide (*n* = 1) and pegvisomant (*n* = 3).

**Figure 4:**
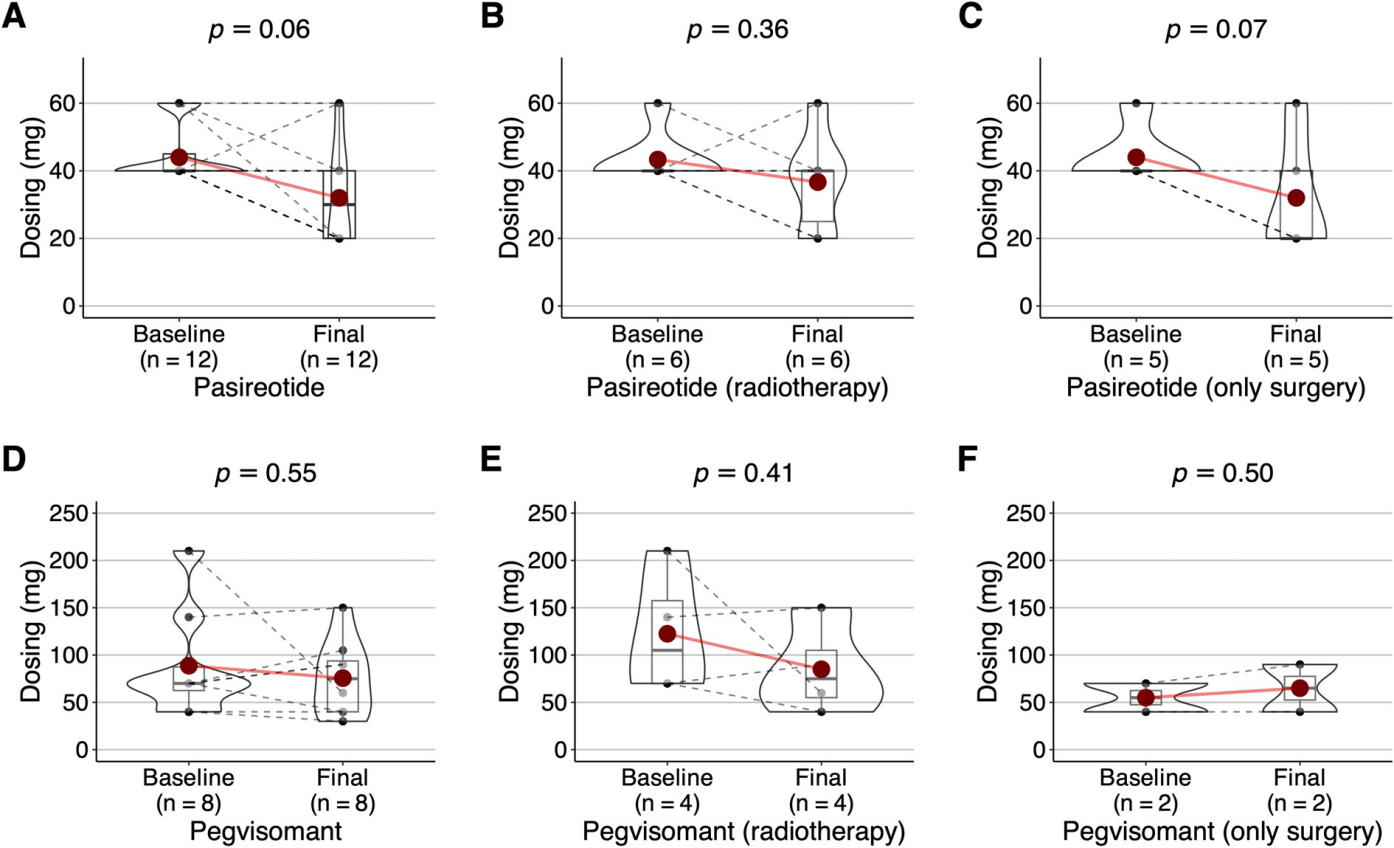
Box and whisker plot (with 10% trimmed mean represented as a reddish dot) with overlapping violin plot and point cloud diagram geometries. Posology (in milligrams) for second-line treatment of acromegaly (pasireotide in mg/month, pegvisomant in mg/week) on the *Y-*axis, treatment time (baseline, final) on the *X*-axis. Results of Yuen’s t-test shown at top. The intraindividual before-after evolution is indicated as solid lines in grey according to the identifier of the participant. All analyses were performed per protocol, including only currently active chronic treatments after excluding participants who discontinued the drug due to side effects or cure. Dose course in the case of pasireotide **(A)** or pegvisomant **(D)**. Dose course in surgery with radiotherapy group treated with pasireotide **(B)** or pegvisomant **(E)**. Dose course in surgery without radiotherapy group treated with pasireotide **(C)** or pegvisomant **(F)**.

A per protocol analysis was performed to compare the starting dose versus the final dose, including only patients on chronic treatment (*n =* 12 pasireotide, *n =* 8 pegvisomant). In the case of pasireotide and regardless of treatment modality, the dose changed from 44.0 (9.1) to 32.0 (15.6) mg/month (*P = .*06) (**Figure 4A**). Although there were no differences in patients with radiotherapy (**Figure 4B**), there was a similar trend towards dose reduction in surgical cases (*P = .*07) (**Figure 4C**). In the case of pegvisomant, the course changed from 88.7 (57.9) to 75.6 (40.8) mg/week (*P* = .55) (**Figure 4D**). No significant before-after differences were found in the radiotherapy (*P* = .41, **Figure 4E**) or surgery (*P* = .50, **Figure 4F**) groups.

### 3.6 Efficacy and safety of second-line drugs for the treatment of acromegaly

IGF-1 control proved adequate with both drugs: from 395.4 (129.5) to 169.4 (54.3) ng/ml (*P* <.001) in the case of pasireotide, and 370.1 (188.5) to 180.0 (102.1) ng/ml (*P* <.001) for pegvisomant. IGF-1 normalization rates applying our laboratory reference values (**Supplementary Table 1**) evolved from 0% to 100.00% in pasireotide (*P* < 0.001), and from 25.00% to 95.00% in pegvisomant (*P* < 0.001).

No major adverse events were found in the case of pegvisomant. There was a single major adverse event in the case of pasireotide: lithiasic cholangitis, requiring *n =* 1 magnetic resonance cholangiopancreatography with and without contrast, *n =* 1 diagnostic endoscopic retrograde cholangiopancreatography (ERCP) and *n =* 1 abdominal ultrasound, with admission *n* = 15 days in Gastroenterology. The patient subsequently relapsed and required *n =* 18 days of admission in Surgery with *n =* 1 laparoscopic cholecystectomy plus biliary tract examination and *n =* 1 diagnostic-therapeutic ERCP due to biliary fistula following cholecystectomy. The total cost of this incident was 21,581.01 €. Currently, the patient has continued treatment with pegvisomant 30 mg twice a week.

In the case of pasireotide, there was a single case of clinically significant deterioration of blood glucose control that was managed on an outpatient basis after drug discontinuation, and *n =* 4 patients treated with pasireotide experienced non-specific abdominal discomfort with no evidence of lithiasic disease. In the case of pegvisomant, there was *n =* 1 worsening of headache and *n =* 1 lipohypertrophy at injection sites.

**Figure 5:**
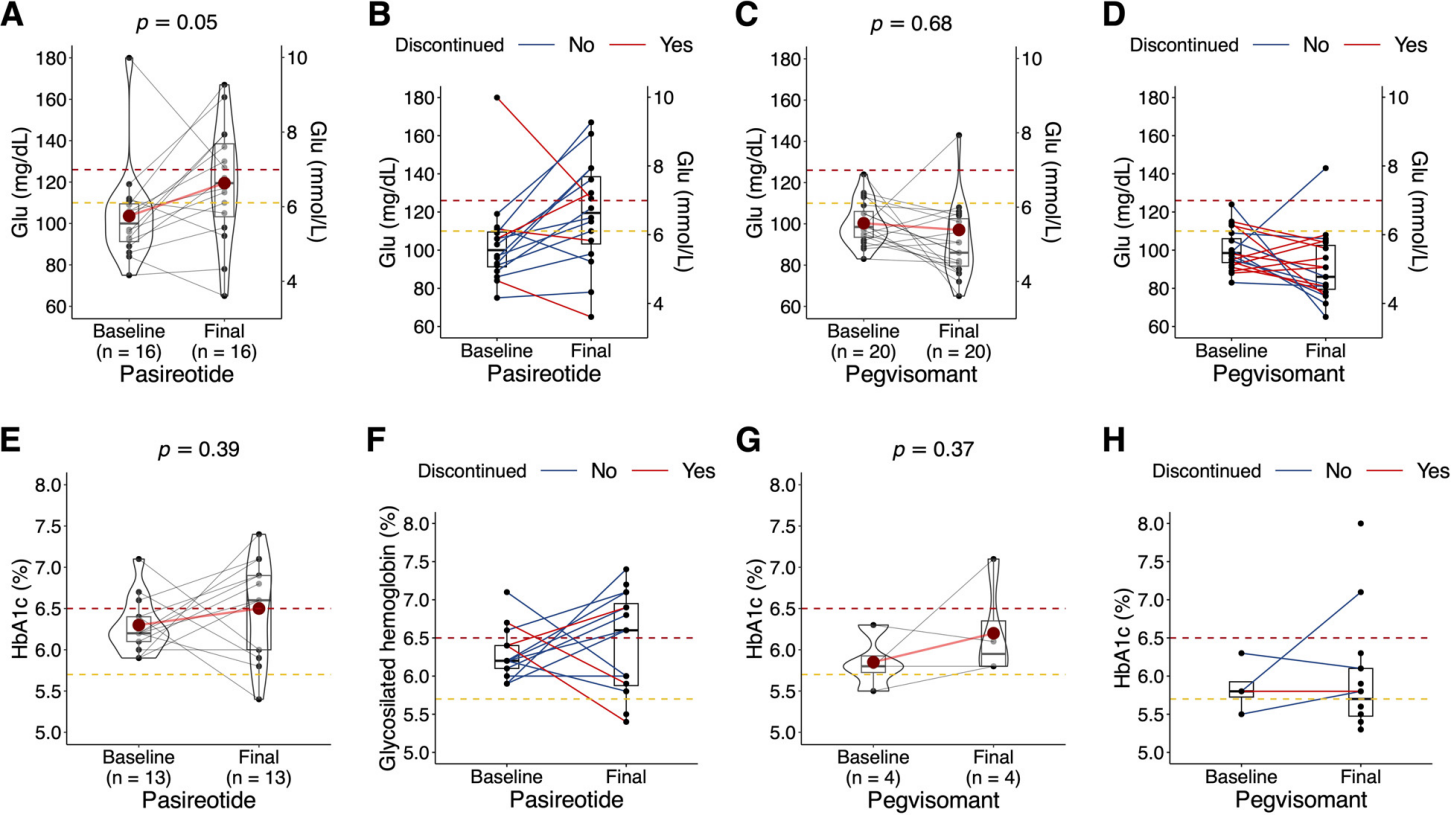
Box and whisker plot (with 10% trimmed mean represented as a reddish dot) with overlapping violin plot and point cloud diagram geometries. Type of second-line treatment for acromegaly: pasireotide (left half of the figure); or pegvisomant (right half of the figure), and time of treatment (baseline, final) on the *X-*axis. Results of Yuen’s t-test shown at top. Fasting plasma glucose (Glu) on the *Y-*axis in the first row **(A–D)** and glycated hemoglobin (HbA1c) on the *Y-*axis in the second row **(E–H)**. Intent-to-treat analysis for pasireotide **(A, E)** and pegvisomant **(C, G)**. Per protocol analysis for pasireotide **(B, F)** and pegvisomant **(D, H)**. Chronicity of the treatment marked in color (blue continued, red discontinued). In all cases, the yellow and red dotted horizontal lines mark the pre-T2DM diagnosis limit and the T2DM diagnosis limit, respectively. Fasting plasma glucose is expressed in both mg/dl (left) and mmol/l (right), while glycated hemoglobin is expressed in percentages (%).

Pasireotide significantly altered the diagnosis of carbohydrate metabolism disorders (*P* = .005), while pegvisomant did not (*P* = .33) (**Table 5**). Both Glu and HbA1c followed a non-normal distribution in the sample. In the case of pasireotide, Glu went from 100.2 (23.5) to 120.0 (27.9) mg/dl (*P* = .05) in ITT analysis (**Figure 5A**), and from 98.3 (12.3) to 124.0 (27.2) mg/dl in PP analysis (*P* <.001) (**Figure 5B**). In the case of pegvisomant, Glu went from 99.7 (10.4) to 90.5 (32.3) mg/dl (*P* = .68) in ITT analysis (**Figure 5C**), and from 101.5 (11.9) to 88.9 (25.0) mg/dl (*P* = .26) in PP analysis (**Figure 5D**). In the case of pasireotide, HbA1c changed from 6.3 (0.3) to 6.4 (0.6) % (*P* = .39) in ITT analysis (**Figure 5E**), and from 6.2 (0.4) to 6.6 (5.7) % (*P* = .13) in PP analysis (**Figure 5F**). In the case of pegvisomant, HbA1c changed from 5.8 (0.3) to 5.9 (0.7) % (*P* = .37) in ITT analysis (**Figure 5G**), and from 5.8 (0) to 5.9 (0.8) % (*P* = .40) in PP analysis (**Figure 5H**).

**Table 5:** Alterations of carbohydrate metabolism in the longitudinal study.

		Baseline	Final
Pasireotide	T2DM (prop, %) Pre-T2DM (prop, %) Normal (prop, %)	3/16 (18.7%) 12/16 (75.0%) 1/16 (6.3%)	13/16 (81.2%) 3/16 (18.8%) 0
Pegvisomant	T2DM (prop, %) Pre-T2DM (prop, %) Normal (prop, %)	1/20 (5.0%) 6/20 (30.0%) 13/20 (65.0%)	4/20 (20.0%) 6/20 (30.0%) 10/20 (50.0%)

T2DM, type 2 diabetes mellitus; pre-T2DM, pre-type 2 diabetes mellitus; prop, proportion.

## 4 Discussion

In our real-life study, pasireotide was found to be a more cost-efficient option for the second-line treatment of acromegaly in situations of failure of first-generation SSAs, particularly in patients treated with high doses of pasireotide. The mean dose of pegvisomant in this study was similar to the Spanish median maintenance dose after 3 to 7 years of treatment in the ACROSTUDY, approximately equivalent to 105 mg/week (18). The cost of pegvisomant at full doses in the study sample was also similar to previous evidence (24). In general, we found the drug cost of acromegaly to be proportional to the aggressiveness of the disease, with more cost in patients that required a second surgical intervention.

Regarding direct costs attributable to the medical and non-medical treatment of acromegaly (including serious adverse events), previous theoretical studies conducted in Spain (16) and in France (25) using probabilistic models based on data from pivotal clinical trials found pegvisomant to be the most cost-effective treatment. In the aforementioned works, pasireotide was a less efficient therapy than pegvisomant monotherapy, not at the expense of a higher cost of the drug, but at a higher cost of the adverse events associated with it. In our study, pasireotide presented one serious adverse event during follow-up, attaining a unit cost lower than one year of treatment with low-dose pasireotide.

Although the addition of cabergoline (in the case of pasireotide and pegvisomant) and/or first-generation SSAs (in the case of pegvisomant) form part of the recommendations on the pharmacological management of acromegaly (14, 26), no dose- or cost-saving effect was found cross-sectionally in our sample. This may be explained by the refractory nature of the included cases, with costly high doses of pasireotide and pegvisomant.

The cost attributable to hormone replacement therapy and T2DM treatment was minimal compared to the cost of the second-line drugs for the treatment of acromegaly. Compared to the total direct drug costs, the excess cost associated to treatment of T2DM was minimal in the case of both pasireotide and pegvisomant. However, the impact of T2DM on the need for care and quality of life should also be taken into consideration.

In the longitudinal study, both drugs were effective. About dose course, pasireotide was associated with a dose reduction in both surgical and irradiated patients. However, the only pegvisomant group that was able to reduce dose (yet without reaching statistical significance) was that of patients who had surgery and radiotherapy.

The potential deterioration of blood glucose control with the use of acromegaly drugs is a concern for both patients (27) and caregivers (28). Although clinically significant, the deterioration of blood glucose control with pasireotide in our research was consistent with previous evidence (29–34) and generally proved mild, unlike in older studies (35). Apart from a potential selection bias for pegvisomant in patients with more risk factors for T2DM or worse metabolic control, the introduction in recent years of GLP1 receptor agonists (36) and SGLT2 inhibitors allows for improving and simplifying metabolic control in these patients, as they are already being monitored by an endocrinology specialist who can coordinate multimodal care for T2DM (37).

Our study has several strengths. As this is a real-life study in a public healthcare setting, it should have sufficient external validity to be applicable in other centers of the Spanish National Health System. Regarding the cohort study, the monographic acromegaly clinic has been managed according to a single internal protocol, which homogenizes the clinical criterion used throughout the whole sample and improves the internal validity of the study.

This work has a number of limitations. Firstly, the retrospective nature of the data increases imprecision and has made it impossible to obtain HbA1c in all cases in the longitudinal study. Although this study has a large sample size in relative terms for a single-center trial and involving an uncommon disease, its sample size is small in absolute terms: this reduces the statistical power, precludes certain sub-analyses, and increases artefacts due to outliers. On the other hand, we have not counted for the indirect healthcare costs of the participants or all the direct costs. However, drug therapy may account for 71% of the direct costs of the disease (38). We therefore believe that although the methodology used for calculating costs is not perfect, it gives us an adequate approximation of the actual direct cost of the participants.

Previous authors have already noted the need to conduct real-life studies to test the efficiency of the different treatment options available for acromegaly in order to optimize resource allocation (39). Treatment individualization is key in patients with refractory acromegaly (40). The cost of treatment is a secondary factor (28), but needs to be taken into account. We believe that the results of this study could be of interest to facilitate resource optimization in other pituitary units in our setting. We propose starting pasireotide in all cases of failure with first-generation analogs, with the use of pegvisomant being left for patients with side effects to somatostatin analogs and those with hard-to-control diabetes.

## Data Availability

The datasets presented in this article are not readily available because the data presented in this study are available on request from the corresponding author as per European legislation on data protection. Requests to access the datasets should be directed to andres.jimenez.sanchez.sspa@juntadeandalucia.es.

## References

[1] Crisafulli S Luxi N Sultana J Fontana A Spagnolo F Giuffrida G. Global epidemiology of acromegaly: a systematic review and meta-analysis. Eur J Endocrinol. (2021) 185:251–63. doi: 10.1530/EJE-21-0216 34061771

[2] Aagaard C Christophersen AS Finnerup S Rosendal C Gulisano HA Ettrup KS. The prevalence of acromegaly is higher than previously reported: Changes over a three-decade period. Clin Endocrinol (Oxf). (2022) 97:773–82. doi: 10.1111/cen.14828 PMC9827885 36163677

[3] Rosendal C Arlien-Søborg MC Nielsen EH Andersen MS Feltoft CL Klose M. Changes in acromegaly comorbidities, treatment, and outcome over three decades: a nationwide cohort study. Front Endocrinol. (2024) 15:1380436. doi: 10.3389/fendo.2024.1380436 PMC11024468 38638137

[4] Xiao Z Xiao P Wang Y Fang C Li Y. Risk of cancer in acromegaly patients: An updated meta-analysis and systematic review. PloS One. (2023) 18:e0285335. doi: 10.1371/journal.pone.0285335 38032888 PMC10688666

[5] Parolin M Dassie F Martini C Mioni R Russo L Fallo F. Preclinical markers of atherosclerosis in acromegaly: a systematic review and meta-analysis. Pituitary. (2018) 21:653–62. doi: 10.1007/s11102-018-0911-5 30225826

[6] Guo X Fu H Pang H Xing B. Risk of left ventricular hypertrophy and diastolic and systolic dysfunction in Acromegaly: A meta-analysis. J Clin Neurosci Off J Neurosurg Soc Australas. (2018) 48:28–33. doi: 10.1016/j.jocn.2017.10.067 29097130

[7] Parolin M Dassie F Alessio L Wennberg A Rossato M Vettor R. Obstructive sleep apnea in acromegaly and the effect of treatment: A systematic review and meta-analysis. J Clin Endocrinol Metab. (2020) 105:dgz116. doi: 10.1210/clinem/dgz116 31722411

[8] Bolfi F Neves AF Boguszewski CL Nunes-Nogueira VS. Mortality in acromegaly decreased in the last decade: a systematic review and meta-analysis. Eur J Endocrinol. (2018) 179:59–71. doi: 10.1530/EJE-18-0255 29764907

[9] Maione L Brue T Beckers A Delemer B Petrossians P Borson-Chazot F. Changes in the management and comorbidities of acromegaly over three decades: the French Acromegaly Registry. Eur J Endocrinol. (2017) 176:645–55. doi: 10.1530/EJE-16-1064 28246150

[10] Gadelha MR Kasuki L Lim DST Fleseriu M. Systemic complications of acromegaly and the impact of the current treatment landscape: an update. Endocr Rev. (2019) 40:268–332. doi: 10.1210/er.2018-00115 30184064

[11] Broersen LHA Zamanipoor Najafabadi AH Pereira AM Dekkers OM van Furth WR Biermasz NR. Improvement in symptoms and health-related quality of life in acromegaly patients: A systematic review and meta-analysis. J Clin Endocrinol Metab. (2021) 106:577–87. doi: 10.1210/clinem/dgaa868 PMC7823264 33245343

[12] Katznelson L Laws ER Melmed S Molitch ME Murad MH Utz A. Acromegaly: an endocrine society clinical practice guideline. J Clin Endocrinol Metab. (2014) 99:3933–51. doi: 10.1210/jc.2014-2700 25356808

[13] Briceno V Zaidi HA Doucette JA Onomichi KB Alreshidi A Mekary RA. Efficacy of transsphenoidal surgery in achieving biochemical cure of growth hormone-secreting pituitary adenomas among patients with cavernous sinus invasion: a systematic review and meta-analysis. Neurol Res. (2017) 39:387–98. doi: 10.1080/01616412.2017.1296653 28301972

[14] Melmed S Bronstein MD Chanson P Klibanski A Casanueva FF Wass JAH. A Consensus Statement on acromegaly therapeutic outcomes. Nat Rev Endocrinol. (2018) 14:552–61. doi: 10.1038/s41574-018-0058-5 PMC7136157 30050156

[15] Leonart LP Ferreira VL Tonin FS Fernandez-Llimos F Pontarolo R. Medical treatments for acromegaly: A systematic review and network meta-analysis. Value Health J Int Soc Pharmacoeconomics Outcomes Res. (2018) 21:874–80. doi: 10.1016/j.jval.2017.12.014 30005760

[16] Peral C Cordido F Gimeno-Ballester V Mir N Sánchez-Cenizo L Rubio-Rodríguez D. Cost-effectiveness analysis of second-line pharmacological treatment of acromegaly in Spain. Expert Rev Pharmacoecon Outcomes Res. (2020) 20:105–14. doi: 10.1080/14737167.2019.1610396 31055976

[17] von Elm E Altman DG Egger M Pocock SJ Gøtzsche PC Vandenbroucke JP. STROBE Initiative. Strengthening the Reporting of Observational Studies in Epidemiology (STROBE) statement: guidelines for reporting observational studies. BMJ. (2007) 335:806–8. doi: 10.1136/bmj.39335.541782.AD PMC2034723 17947786

[18] Grottoli S Bianchi A Bogazzi F Bona C Carlsson MO Colao A. Are there country-specific differences in the use of pegvisomant for acromegaly in clinical practice? An analysis from ACROSTUDY. J Endocrinol Invest. (2022) 45:1535–45. doi: 10.1007/s40618-022-01789-4 PMC9270309 35359232

[19] Precios públicos. Serv andal salud . Available online at: https://www.sspa.juntadeandalucia.es/servicioandaluzdesalud/profesionales/relacion-con-la-ciudadania/precios-publicos (Accessed April 4, 2024).

[20] Wilke CO. cowplot: streamlined plot theme and plot annotations for “ggplot2.” (2024). Available online at: https://cran.r-project.org/web/packages/cowplot/index.html (Accessed November 2, 2024).

[21] Kassambara A. ggpubr: “ggplot2” Based publication ready plots (2023). Available online at: https://cran.r-project.org/web/packages/ggpubr/index.html (Accessed November 2, 2024).

[22] Patil I Powell C. ggstatsplot: “ggplot2” Based plots with statistical details (2024). Available online at: https://cran.r-project.org/web/packages/ggstatsplot/index.html (Accessed November 26, 2024).

[23] Wickham H Averick M Bryan J Chang W McGowan LD François R. Welcome to the tidyverse. J Open Source Softw. (2019) 4:1686. doi: 10.21105/joss.01686

[24] Bernabeu I Rodriguez-Gomez IA Ramos-Levi AM Marazuela M. Profile of pegvisomant in the management of acromegaly: an evidence based review of its place in therapy. Res Rep Endocr Disord. (2015) 5:47–58. doi: 10.2147/RRED.S78255

[25] Brue T Chanson P Rodien P Delemer B Drui D Marié L. Cost-utility of acromegaly pharmacological treatments in a french context. Front Endocrinol. (2021) 12:745843. doi: 10.3389/fendo.2021.745843 PMC8531881 34690933

[26] Coopmans EC Muhammad A van der Lely AJ Janssen JAMJL Neggers SJCMM. How to position pasireotide LAR treatment in acromegaly. J Clin Endocrinol Metab. (2019) 104:1978–88. doi: 10.1210/jc.2018-01979 30608534

[27] Fajardo C Álvarez-Escola C Biagetti B Garcia-Centeno R Ciriza R Sánchez-Cenizo L. Preference of acromegaly patients for treatment attributes in Spain. Endocrine. (2023) 82:379–89. doi: 10.1007/s12020-023-03462-z PMC10543785 37507554

[28] Grottoli S Maffei P Tresoldi AS Granato S Benedan L Mariani P. Insights from an Italian Delphi panel: exploring resistance to first-generation somatostatin receptor ligands and guiding second-line medical therapies in acromegaly management. J Endocrinol Invest. (2024) 47:2999–3017. doi: 10.1007/s40618-024-02386-3 38809458 PMC11549125

[29] Muhammad A Coopmans EC Delhanty PJD Dallenga AHG Haitsma IK Janssen JAMJL. Efficacy and Safety of switching to Pasireotide in Acromegaly Patients controlled with Pegvisomant and Somatostatin Analogues: PAPE extension study. Eur J Endocrinol. (2018) 179:269–77. doi: 10.1530/EJE-18-0353 30076159

[30] Bhat SZ Salvatori R. Current role of pasireotide in the treatment of acromegaly. Best Pract Res Clin Endocrinol Metab. (2024) 38, 101875. doi: 10.1016/j.beem.2024.101875 38290866

[31] Störmann S Meyhöfer SM Groener JB Faust J Schilbach K Seufert J. Management of pasireotide-induced hyperglycemia in patients with acromegaly: An experts’ consensus statement. Front Endocrinol. (2024) 15:1348990. doi: 10.3389/fendo.2024.1348990 PMC10884330 38405148

[32] Roset M Merino-Montero S Luque-Ramírez M Webb SM López-Mondéjar P Salinas I. Cost of clinical management of acromegaly in Spain. Clin Drug Investig. (2012) 32:235–45. doi: 10.2165/11599680-000000000-00000 22397307

[33] Kamusheva M Rusenova Y Vandeva S Elenkova A Zaharieva S Doneva M. Economic and pharmaco-economic analysis of acromegaly treatment: a systematic review. Biotechnol Equip. (2019) 33:1560–71. doi: 10.1080/13102818.2019.1680317

[34] Chiloiro S Giampietro A Mirra F DonFrancesco F Tartaglione T Mattogno PP. Pegvisomant and Pasireotide LAR as second line therapy in acromegaly: clinical effectiveness and predictors of response. Eur J Endocrinol. (2021) 184:217–29. doi: 10.1530/EJE-20-0767 33136550

[35] Corica G Pirchio R Milioto A Nista F Arecco A Mattioli L. Pasireotide effects on biochemical control and glycometabolic profile in acromegaly patients switched from combination therapies or unconventional dosages of somatostatin analogs. J Endocrinol Invest. (2024) 47:683–97. doi: 10.1007/s40618-023-02186-1 37695461

[36] Gadelha M Marques NV Fialho C Scaf C Lamback E Antunes X. Long-term efficacy and safety of pasireotide in patients with acromegaly: 14 years of single-center real-world experience. J Clin Endocrinol Metab. (2023) 108:e1571–9. doi: 10.1210/clinem/dgad378 PMC10655523 37357993

[37] Urbani C Dassie F Zampetti B Mioni R Maffei P Cozzi R. Real-life data of Pasireotide LAR in acromegaly: a long-term follow-up. J Endocrinol Invest. (2024) 47:1733–41. doi: 10.1007/s40618-023-02275-1 PMC11196287 38244140

[38] Witek P Bolanowski M Szamotulska K Wojciechowska-Luźniak A Jawiarczyk-Przybyłowska A Kałużny M. The effect of 6 months’ Treatment with pasireotide LAR on glucose metabolism in patients with resistant acromegaly in real-world clinical settings. Front Endocrinol. (2021) 12:633944. doi: 10.3389/fendo.2021.633944 PMC7988223 33776927

[39] Yedinak CG Hopkins S Williams J Ibrahim A Cetas JS Fleseriu M. Medical therapy with pasireotide in recurrent cushing’s disease: experience of patients treated for at least 1 year at a single center. Front Endocrinol. (2017) 8:35. doi: 10.3389/fendo.2017.00035 PMC5327352 28289402

[40] Favero V Zampetti B Carioni EI Dalino Ciaramella P Grossrubatscher E Dallabonzana D. Efficacy of pasireotide LAR for acromegaly: a prolonged real-world monocentric study. Front Endocrinol. (2024) 15:1344728. doi: 10.3389/fendo.2024.1344728 PMC10867143 38362280

