# Supplementary material for: Cost-effectiveness analysis of second-line medical therapies in acromegaly: a real-life study
Source: Front Endocrinol (Lausanne). 2025 Apr 28;16:1573721. doi: 10.3389/fendo.2025.1573721 (PMC12066263; doi:10.3389/fendo.2025.1573721)
Supplement: Supplementary file 1 [file Table1.docx]

# Supplementary Figures and Tables

## Supplementary Tables

**Supplementary Table 1.** Reference values for IGF-1 in our laboratory.

| Age | IGF-1 (ng/ml) |
| --- | --- |
| 19 to 20 years | 127 to 483 |
| 21 to 25 years | 116 to 358 |
| 26 to 40 years | 109 to 329 |
| 41 to 55 years | 87 to 267 |
| > 55 years | 55 to 225 |

**Supplementary Table 2.** Cost of hormone replacement therapy.

| Drug | Dose per unit | Units per container | Cost per container (€) | Cost per unit (€)* |
| --- | --- | --- | --- | --- |
| Transdermal estradiol (several brands) | 50 μg | 8 | 6.92 | 0.86 |
| Progesterone capsules (several brands) | 100 mg | 30 | 5.81 | 0.19 |
| Reandron® (Bayer HealthCare Pharmaceuticals) | 1,000 mg | 1 | 89.61 | 89.61 |
| Itnogen® 20 mg/g (2%) gel 60 g (ProStrakan) | 1,200 mg | 1 | 42.34 | 0.02 |
| Testex prolongatum® (Desma) | 250 mg / 2 ml | 1 | 8.65 | 8.65 |
| Testex prolongatum® (Desma) | 100 mg / 2 ml | 1 | 5.12 | 5.12 |
| Eutirox® (Merck) | 50 μg | 50 | 2.79 | 0.09 |
| Eutirox® (Merck) | 75 μg | 75 | 4.09 | 0.14 |
| Eutirox® (Merck) | 88 μg | 88 | 4.51 | 0.15 |
| Eutirox® (Merck) | 100 μg | 100 | 4.93 | 0.16 |
| Eutirox® (Merck) | 112 μg | 112 | 5.18 | 0.17 |
| Eutirox® (Merck) | 125 μg | 125 | 5.70 | 0.19 |
| Eutirox® (Merck) | 137 μg | 137 | 5.85 | 0.20 |
| Eutirox® (Merck) | 150 μg | 150 | 6.46 | 0.22 |
| Eutirox® (Merck) | 175 μg | 175 | 7.09 | 0.24 |
| Eutirox® (Merck) | 200 μg | 200 | 7.68 | 0.26 |
| Minurin® Flas (Ferring Pharmaceuticals) | 120 μg | 30 | 34.16 | 1.14 |
| Hidroaltesona® (Alter) | 20 mg | 30 | 3.12 | 0.10 |
| * Cost per dose in the case of testosterone gel in tube | | | | |

**Supplementary Table 3.** Cost of drugs for type 2 diabetes mellitus.

| Drug | Dose per unit | Units per container** | Cost per container (€) | Cost per unit (€)*** |
| --- | --- | --- | --- | --- |
| Empagliflozin: Jardiance® (Boehringer Ingelheim) | 25 mg | 30 | 51.52 | 1.72 |
| Gliclazide (generic) | 30 mg | 60 | 5.59 | 0.09 |
|  | 60 mg |  | 11.18 | 0.19 |
| Insulin glargine 100 IU/ml: Lantus® (Sanofi) | 300 IU | 5 x 3.0 ml | 56.25 | 0.04 |
| Insulin glargine 200 IU/ml: Toujeo® Solostar (Sanofi) | 300 IU | 3 x 1.5 ml | 50.63 | 0.06 |
| Insulin aspart 100 IU/ml: Novorapid® (NovoNordisk) | 300 IU | 5 x 3.0 ml | 43.21 | 0.03 |
| Metformin (generic) | 850 mg | 50 | 1.94 | 0.06 |
|  | 1,000 mg | 30 | 1.86 | 0.06 |
| Metformin / alogliptin: Vipdomet® (Takeda Pharma, Menarini) | 850 mg / 12.5 mg | 56 | 48.96 | 0.87 |
| Linagliptin / empagliflozin: Glyxambi® (Boehringer Ingelheim) | 10 mg / 5 mg | 30 | 92.26 | 3.07 |
| Metformin / canagliflozin: Vokanamet® (Janssen) | 1,000 mg / 150 mg | 60 | 83.83 | 1.40 |
| Metformin / dapagliflozin: Xigduo® (AstraZeneca) | 850 mg / 5 mg | 56 | 49.83 | 0.89 |
| Metformin / empagliflozin (Synjardy®) | 1,000 mg / 5 mg | 60 | 53.39 | 0.89 |
| Metformin / sitagliptin (generic) | 1,000 mg / 100 mg | 28 | 28.10 | 1.00 |
| Repaglinide (generic) | 1 mg | 90 | 5.59 | 0.06 |
| Oral semaglutide (Rybelsus®) | 7 mg | 30 | 132.77 | 4.43 |
|  | 14 mg |  | 132.77 | 4.43 |
| Subcutaneous semaglutide (Ozempic®) | 0.5 mg | 1 | 128.15 | 128.15 |
|  | 1 mg |  | 128.15 | 128.15 |
| Sitagliptin (generic) | 100 mg | 28 | 31.21 | 1.04 |
|  | 50 mg |  | 16.78 | 0.56 |
| Contour Next® capillary blood glucose strip (Bayer HealthCare Pharmaceuticals) | NA | 50 | 14.72 | 0.90 |
| NA: Not applicable; * IU per ml in the case of insulins ** units: tablets / injectables / strips *** cost per IU in the case of insulins | | | | |

**Supplementary Table 4.** IGF-1 normalization rates with second line acromegaly treatment in the longitudinal study.

|  |  | **Baseline** | **Final** |
| --- | --- | --- | --- |
| Pasireotide | Controlled (prop, %) | 0/16 | 16/16 (100.00 %) |
|  | Not controlled (prop, %) | 16/16 (100.00 %) | 0/16 |
| Pegvisomant | Controlled (prop, %) | 5/20 (25.00 %) | 19/20 (95.00 %) |
|  | Not controlled (prop, %) | 15/20 (75.00 %) | 1/20 (5.00 %) |

prop: proportion
